# Resistin Expression in Epithelial Ovarian Cancer promotes the Proliferation and Migration of Ovarian Cancer Cells to Worsen Prognosis

**DOI:** 10.7150/jca.62496

**Published:** 2021-09-23

**Authors:** Li Pang, Xiaohan Chang

**Affiliations:** Department of Obstetrics and Gynecology, ShengJing Hospital of China Medical University, Shenyang, Liaoning, China.

**Keywords:** Gynecological cancer, rapamycin, mTOR, P70S6K, obesity, FIGO

## Abstract

**Background:** Epithelial ovarian cancer (EOC) is the most common gynecological cancer in women. Resistin, an inflammatory adipocytokine, is associated with obesity, insulin resistance, and various cancer types**.**

**Materials and Methods:** We investigated resistin expression in tissues and its association with the clinicopathological characteristics and prognosis of patients with EOC. The SKOV3 and CAOV3 cell lines were treated with exogenous resistin and rapamycin (resistin inhibitor), and the expression of mTOR in SKOV3 and CAOV3 cells was measured. Cell proliferation was measured using the CCK-8 assay. Western blotting analysis was performed to examine the phosphorylation of P70S6K and mTOR. Wound healing and Transwell analyses were conducted to examine the effect of resistin on the migration of SKOV3 and CAOV3 cells.

**Results:** High resistin expression was positively correlated with the pathological grade (*P* = 0.017) and lymph node metastasis (*P* = 0.045). However, resistin expression was not correlated with age, FIGO stage, or residual tumor after initial laparotomy (*P* > 0.05). Cox multivariate analysis showed that resistin expression was an independent factor for determining disease-free survival, whereas lymph node metastasis, resistin expression, and age (≥55 years) were independent factors affecting overall survival. Exogenous resistin induced ovarian cancer cell proliferation, whereas rapamycin had the opposite effect. Resistin promoted the proliferation of ovarian cancer cells via the mTOR signaling pathway and was associated with phosphorylating P70S6K. Furthermore, resistin promoted the migration of ovarian cancer cells.

**Conclusions:** Resistin may promote the occurrence of ovarian cancer and is related to the prognosis of patients. This protein may also affect the proliferation of EOC cells through the mTOR signaling pathway. Therefore, resistin shows potential as a molecular therapeutic target in ovarian cancer.

## Introduction

Ovarian cancer is the leading cause of gynecological cancer-related death among women. Epithelial ovarian cancer (EOC) is a common malignant ovarian tumor with a 5-year survival rate of <30%. Many factors regulate the rapid growth of EOC. Despite efforts to improve the diagnosis rate and advances in surgical and chemotherapy treatment strategies, ovarian cancer remains difficult to treat because of its rapid growth. Several studies have confirmed an association between obesity and cancer. Body mass index (BMI) was found to be positively correlated with mortality in many cancer types, accounting for 20% of cancer-related deaths in women 1, 2. In addition, studies have shown that obesity can increase the risk of developing endometrial cancer and breast cancer in postmenopausal women and may increase the risk of developing kidney, colon, and gall bladder cancer 3. Although there is no clear evidence of the association between obesity and ovarian cancer prognosis, a recent meta-analysis suggested that BMI is positively correlated with mortality in patients with early ovarian cancer 3. This may be because of the secretion of cytokines by adipose tissue, which in turn may affect the proliferation, apoptosis, invasive growth, and angiogenesis of tumor cells 2-4.

Resistin is an inflammatory adipokine discovered in 2001 as a target of thiazolidinedione. It is mainly secreted by adipose tissue and is involved in insulin resistance, and thus may be related to obesity and diabetes 4-6. Previous studies of resistin mainly focused on its inflammatory and insulin resistance effects. Administration of recombinant resistin in normal mice resulted in the development of insulin resistance. In contrast, administration of resistin-neutralizing antibodies in a mouse model of obesity and insulin resistance led to significant improvements in sensitivity to insulin 4,5. In humans, resistin is expressed not only by adipocytes 7 but also by peripheral blood mononuclear cells 8. Studies have shown that resistin is related to uterine fibroids, polycystic ovary syndrome, and choriocarcinoma. It is also closely related to the malignant biological behaviors of colon, liver, oral squamous, lung adenocarcinoma, gastric, and breast cancer 9-14.

In this study, we investigated resistin expression in EOC tissues by immunohistology and analyzed the possible correlation between resistin expression and clinicopathological characteristics and lymph node (LN) metastasis in patients with EOC. We also analyzed the correlation between resistin expression and patient prognoses based on the clinical characteristics of the patients. We further examined the effect of resistin on the proliferation and migration of ovarian cancer cells (SKOV3 and CAOV3 cells) and its primary mechanism of action. These findings provide a new approach for the diagnosis and treatment of ovarian cancer.

## Material and methods

### Chemicals, reagents, and equipment

Two human ovarian epithelial carcinoma cell lines (CAOV3 and SKOV3) were purchased from ATCC (Manassas, VA, USA). McCoy5A medium was purchased from HyClone (Logan, UT, USA). Recombinant resistin, mTOR antibody, rapamycin, adult fat mesenchymal stem cells, and adult fat mesenchymal stem cells complete medium were purchased from Phoenix Pharmaceuticals (Phoenix Pharmaceuticals, Mannheim, Germany). Anti-βactin antibody was obtained from Keygen Biotech (1:1000, Nanjing, China). Phospho (p)- mTOR, mTOR, p- p70S6 kinase (Thr389), and p70S6 kinase (Thr389) were purchased from Cell Signaling Technology (1:1000, Danvers, MA, USA). Cell Counting Kit-8 (CCK-8) was supplied by Dojindo (Kumamoto, Japan), and the ELISA plate reader was obtained from Bio-Rad (Hercules, CA, USA).

### Cell culture

SKOV3 or CAOV3 cells were cultured in McCoy5A, containing 10% fetal bovine serum (FBS) and penicillin/streptomycin (100 U/mL), in a humidified incubator at 37 °C. After reaching confluence, the cells were treated with resistin (10-40 ng/mL) for 24-72 h. In addition, some of the cells were pretreated with resistin (10-40 ng/mL) and rapamycin (10, 20, and 30 nM) for 24-72 h.

### Tissue samples

Surgical specimens of EOC (n = 50) were obtained from patients admitted to the Shengjing Affiliated Hospital of China Medical University from 2012-2017 and used for immunohistochemical analysis. The inclusion criteria are as follows: chemotherapy or radiotherapy was not performed before surgery, both procedures were confirmed by two pathologists after surgery, and the pathology was epithelial ovarian cancer; the exclusion criteria include adjuvant therapy (chemotherapy and radiotherapy) before surgery, metastatic cancer, and non-primary ovarian cancer. Clinical data were obtained from clinical databases, and tumor staging was performed according to the International Federation of Gynecology and Obstetrics (FIGO, 2009) guidelines. This study was approved by the institutional review board, and all patients provided written informed consent to participate in the study.

### Immunohistochemical staining and evaluation

Six slices of each tissue sample (1 for hematoxylin and eosin staining, 3 for immunohistochemistry, and 2 as standby) were successively sectioned (4 µm thick) in each row of a wax block. The immunohistochemistry SP (streptavidin-peroxidase) method was used to evaluate the samples. Using the double-blind reading method, resistin expression on tumor cell cytoplasm showing brown-yellow particles was set as a positive determination criterion. The total score was calculated as the sum of the staining intensity (0 = no coloring, 1 = light brown, 2 = tan, 3 = brown) and number of positive cells (0 = no positive cells, 1 = number of positive cells <25%, 2 = positive cells was 25-50%, 3 = number of positive cells >50%). A score of ≤1 was considered as (-), 2-3 as (+), 4-5 as (++), and >5 as (+++) 3. Positive results in this experiment were referred to as (+, ++, or +++).

### Cell proliferation assay (CCK-8)

A cell-free medium was used as the blank control group. In the experimental group, resistin (10-40 ng/mL) and rapamycin 30 nM were added and incubated for 24, 48, and 72 h at 37 °C. After 24 h of incubation, the medium was aspirated, and 10 mL of CCK-8 solution was added to each well. After incubating the culture for another 3 h, a microplate reader was used to detect the absorbance of the samples at 450 nm. The cell proliferation rate was calculated by measuring the optical density of the cell culture at 450 nm and plotting a growth curve.

### Wound healing assay

SKOV3 or CAOV3 cells were seeded in 6-well plates at a density of 1 × 10^6^/well, followed by treatment with resistin. After 12 h of treatment, a sterile microtubule tip was used to scratch the cell monolayer. At 12 h after scratching, the cells were washed twice with fresh medium and observed under an inverted microscope (IX71; Olympus, Tokyo, Japan). The wound healing rate was estimated by measuring the distance between the wound boundaries.

### Adipose cell culture

Adult adipose-derived mesenchymal stem cells were seeded into a six-well plate at a cell density of 2 × 10^4^ cells/cm^2^, and 2 mL of complete medium was added to each well. After cell fusion reached 100%, the mesenchymal stem cells were carefully removed from the complete culture medium, and 2 mL of adult fat mesenchymal stem cell adipogenic differentiation medium (solution A) was added. After 3 days of induction, solution A was aspirated, and 2 mL of the adult adipose-derived mesenchymal stem cell adipogenic differentiation medium (solution B) was added. After 24 h, solution B was aspirated and replaced with solution A for induction. The procedure was repeated five times (20 days). Then, the culture was grown for another 3 days with solution B until the lipid droplets became large and round.

### Matrigel invasion assay

Serum-free RPMI 1640 culture medium and Matrigel gel were diluted (1:5), and the upper surface of each Transwell chamber was coated with 100 µL of this solution. The plates were dried overnight in a 37 °C incubator and set aside. SKOV3 or CAOV3 ovarian cancer cells were cultured in McCoy5A containing 10% FBS and supplemented with different concentrations of rapamycin for 24 h. Thereafter, cells in the logarithmic phase were exposed to trypsin-mediated disruption to prepare a serum-free cell suspension. Then, each chamber was inoculated with 200 μL of the cell suspension (2.5 × 10^5^/mL), and 500 μL of the RPMI 1640 culture (containing 20% FBS) was added to the lower chamber. The solution was incubated at 37 °C for 24 h, after which the cells were removed and washed. Total protein was extracted from the tissues and cells using radioimmunoprecipitation assay buffer to precipitate the protease inhibitors. The number of migrating cells in three random fields was measured. Each experiment was repeated three times.

### Western blotting

Total protein was extracted from the tissues and cells using radioimmunoprecipitation assay buffer to remove protease inhibitors. The protein concentration was determined using a BCA protein detection kit. Total protein was separated by 10% SDS-PAGE and transferred to polyvinylidene fluoride membrane. Tris-buffered saline with Tween solution containing 5% skim milk was used for blocking for 2 h, and the membranes were incubated overnight with a specific primary antibody at 4 °C. The next morning, the corresponding secondary antibody was added, and the sample was incubated at 26 °C for 2 h. The C300 imaging system (Azure Biosyne, Dublin, CA, USA) with an enhanced chemiluminescence method was performed to determine the strength of the protein bands. The relative integral density was determined using ImageJ software (NIH, Bethesda, MD, USA) in comparison with β-actin. The primary antibodies included phospho- p70S6K, p70S6K, and phospho-mTOR, mTOR, and anti-β-actin. We also used a horseradish peroxidase-conjugated secondary antibody. All tests were performed in triplicate.

### Statistical analysis

Data were analyzed with SPSS version 20.0 statistical software (SPSS, Inc. Chicago, IL, USA). Spearman correlation test was used for statistical evaluation of resistin expression and clinicopathological parameters. All data were expressed as the mean ± standard deviation. Overall survival (OS) and disease-free survival (DFS) curves were generated using the Kaplan-Meier method, and differences between curves were assessed using the log-rank test. A Cox proportional hazard model was used to determine the factors related to survival time. A *P*-value < 0.05 was considered statistically significant.

## Results

### Clinical role of resistin expression in ovarian carcinomas

**Figure 1:**
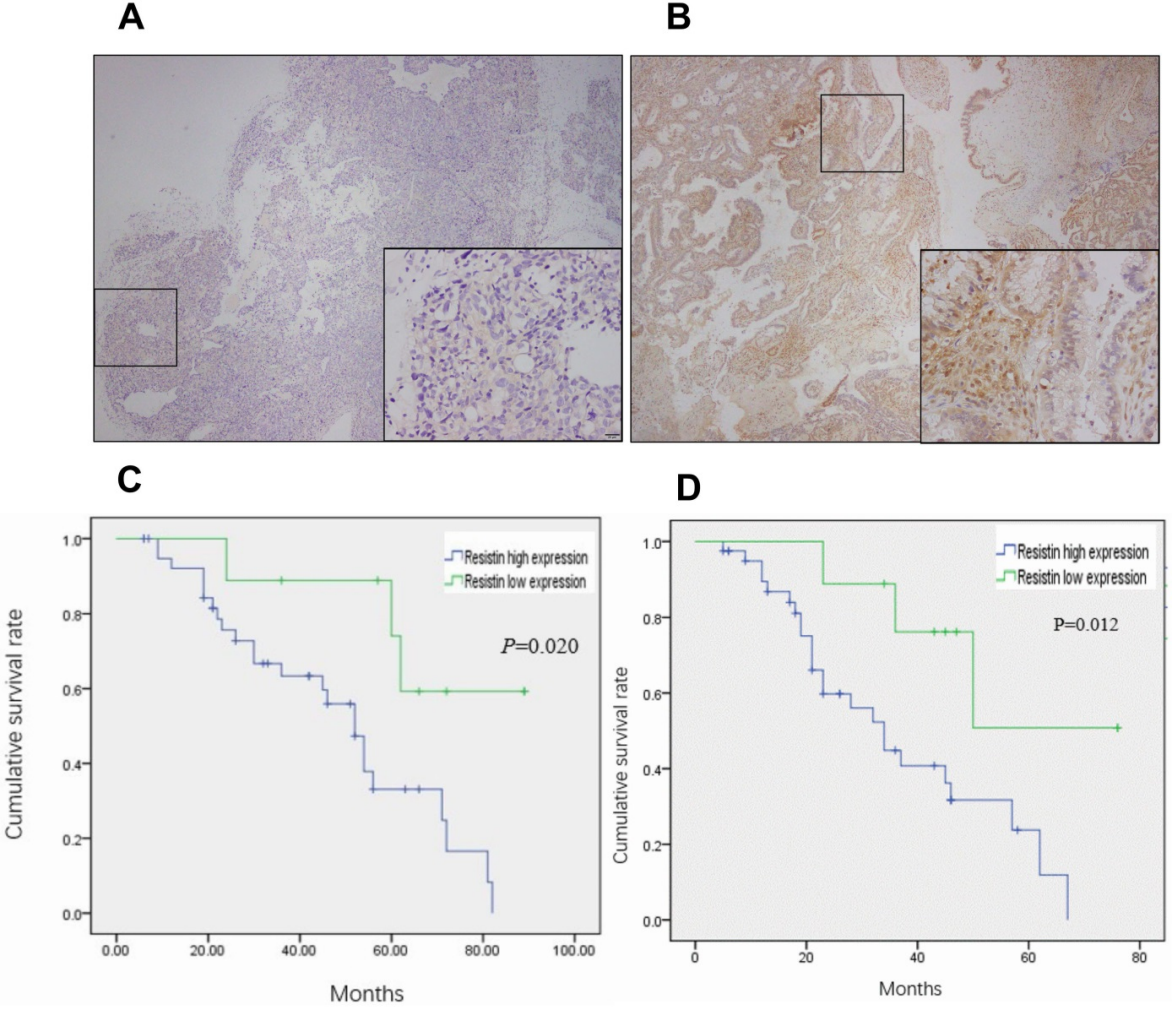
** Resistin expression in epithelial ovarian cancer (EOC) samples and correlation between resistin status and prognosis of patients with EOC. (A)** Normal ovarian tissue and (**B**) immunohistochemical analysis of resistin expression in EOC. **(C)** Kaplan-Meier analysis of disease-free survival (DFS) for patients with EOC according to the resistin expression status. **(D)** Kaplan-Meier analysis of overall survival (OS) for patients with EOC according to the resistin expression status.

A total of 50 EOC specimens, which met the set eligibility criteria, were used in this study. The median age of the patients was 51 years (range: 34-76 years). The number of specimens in FIGO I, II, III, and IV stages were 17, 4, 27, and 2, respectively. The level of cancer antigen 125 ranged from 211-4216 µg/mL (median = 1826.4). Resistin was expressed in only two normal ovarian tissue samples (Figure 1A) and mainly expressed in the cytoplasm and membrane of the EOC specimens (Figure 1B) (*P* < 0.05). As shown in Table 1, resistin expression was associated with pathological grade (*P* = 0.017) and LN metastasis (*P* = 0.045) but not with age, histotype, residual tumor after initial laparotomy, serum cancer antigen 125, and FIGO stage. The EOC survival curves were stratified according to resistin expression. Based on Kaplan-Meier analysis, both OS (*P* = 0.012) and DFS (*P* = 0.020) were related to the resistin expression status (Figure 1C and 1D). Univariate analysis showed that FIGO staging, pathological grade, residual tumor, and LN metastasis were significantly related to DFS and OS (*P* < 0.05, Table 2). Cox multivariate analysis showed that resistin expression was an independent factor affecting OS, and LN metastasis, resistin expression, and age (≥55 years) were independent factors affecting DFS (*P* < 0.05, Table 3).

### Resistin promotes the proliferation of SKOV3 and CAOV3 cells, and rapamycin may reverse these effects

**Figure 2:**
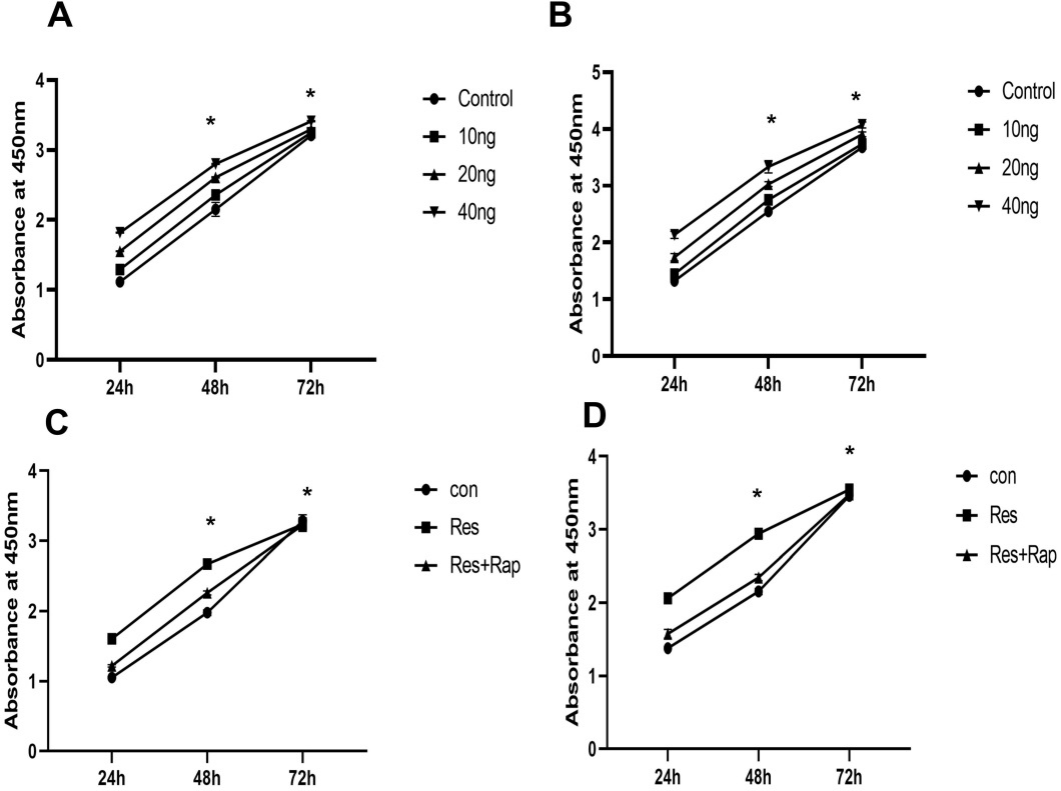
** Effect of resistin on the proliferation of SKOV3 and CAOV3 cells in ovarian epithelial carcinoma cells. (A,B)** Time and dose course of 10-40 ng effect resistin on the proliferation of SKOV3 and CAOV3 cells (*P < 0.05 vs resistin and control groups). **(C,D)** Effect of 30 nM rapamycin treatment on the proliferation of SKOV3 and CAOV3 cells. Rapamycin reduced SKOV3 and CAOV3 cell proliferation, but it did not completely inhibit their growth (*P < 0.05 vs resistin + rapamycin and control group).

Although receptors for resistin have not been identified, mTOR is associated with a variety of cell surface receptor types and proliferation and has been considered to be activated. We investigated whether mTOR activation is involved in the resistin response. The results showed that resistin (10-40 ng) promoted the proliferation of SKOV3 and CAOV3 cells in time- and dose-dependent manners for 24 h. Therefore, in our subsequent experiments, the proliferation rate was higher when measured at 48 and 72 h (Figure 2A and 2B) (*P < 0.05). Resistin and rapamycin induced the proliferation of ovarian cancer cells (SKOV3 and CAOV3) at different time points (24, 48, 72 h). We used CCK-8 to detect whether resistin and rapamycin could promote or inhibit the proliferation rate of SKVO3 and CAOV3 cells. The results demonstrated that rapamycin partly reversed the effects of resistin (Figure 2C and 2D). Based on these results, resistin appears to promote the proliferation of ovarian cancer cells through the mTOR signaling pathway. However, other pathways may also be involved.

### Resistin-induced proliferation of SKOV3 and CAOV3 cells is mediated via the mTOR signaling pathway

**Figure 3:**
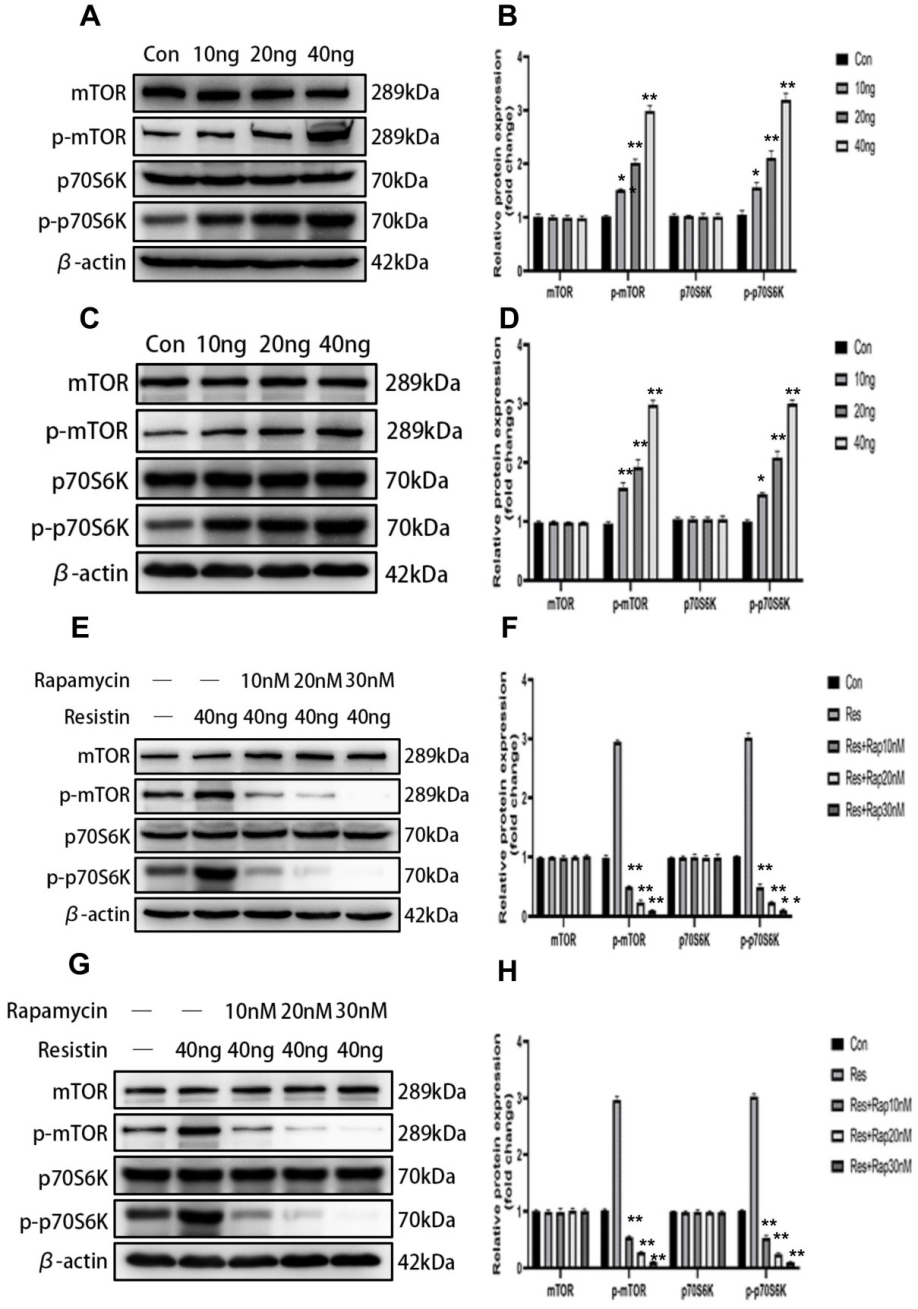
** The mTOR signaling pathway is involved in resistin-mediated induction of SKOV3 and CAOV3 cell proliferation. (A, B)** After resistin stimulation (10-40 ng), the phosphorylation of mTOR and P70S6K was significantly elevated in SKOV3. **P < 0.01 vs p-mTOR and p-P70S6k (resistin 20, 40 ng) and control groups; *P < 0.05 vs p-mTOR and p-P70S6k (resistin 10 ng) and control groups. **(C, D)** After resistin stimulation (10-40 ng), the phosphorylation of mTOR and P70S6K was significantly elevated in CAOV3. **P < 0.01 vs p-mTOR (resistin 10, 20, 40 ng) and control groups, vs p-P70S6k (resistin 20,40 ng) and control groups; *P < 0.05 vs p-P70S6k (resistin 10 ng) and control groups. **(E, F)** Effect of different rapamycin and resistin concentrations on SKOV3 cells after 24h: the phosphorylation of mTOR and P70S6K was significantly reduced. **P < 0.01 vs P-mTOR and p-P70S6k (resistin + 10, 20, 30 nM Rapamycin) and control groups. **(G, H)** Effect of different rapamycin and resistin concentrations on CAOV3 cells after 24 h: the phosphorylation of mTOR and P70S6K was significantly reduced. **P < 0.01 vs P-mTOR and p-P70S6k (resistin + 10, 20, 30 nM Rapamycin) and control groups.

The mTOR signaling pathway is associated with cell proliferation and is considered antiapoptotic. To investigate whether mTOR activation is involved in the resistin response, we performed western blotting to detect the phosphorylation of mTOR and the downstream target protein P70S6K in SKOV3 and CAOV3 cells. After resistin stimulation, the phosphorylation of mTOR and P70S6K was significantly increased. Resistin (10-40 ng) enhanced the phosphorylation of mTOR and P70S6K in a dose-dependent manner for 24 h in SKOV3 (Figure 3A, 3B) and CAOV3 cells (Figure 3C, 3D) (*P < 0.05, **P < 0.01, respectively). Rapamycin is a macrolide immunosuppressant that inhibits mTOR. After SKOV3 and CAOV3 cells were cultured with resistin at a concentration of 40 ng for 24 h, we applied different concentrations of rapamycin (10, 20, and 30 nM). Western blotting was performed to evaluate the expression of mTOR in ovarian cancer cells (SKOV3 and CAOV3). We found that 10-30 nM rapamycin inhibited mTOR expression in SKOV3 (Figure 3E, 3F) and CAOV3 cells (Figure 3G, 3H) (**P < 0.01).

### Resistin promotes the migration of EOC cells

**Figure 4:**
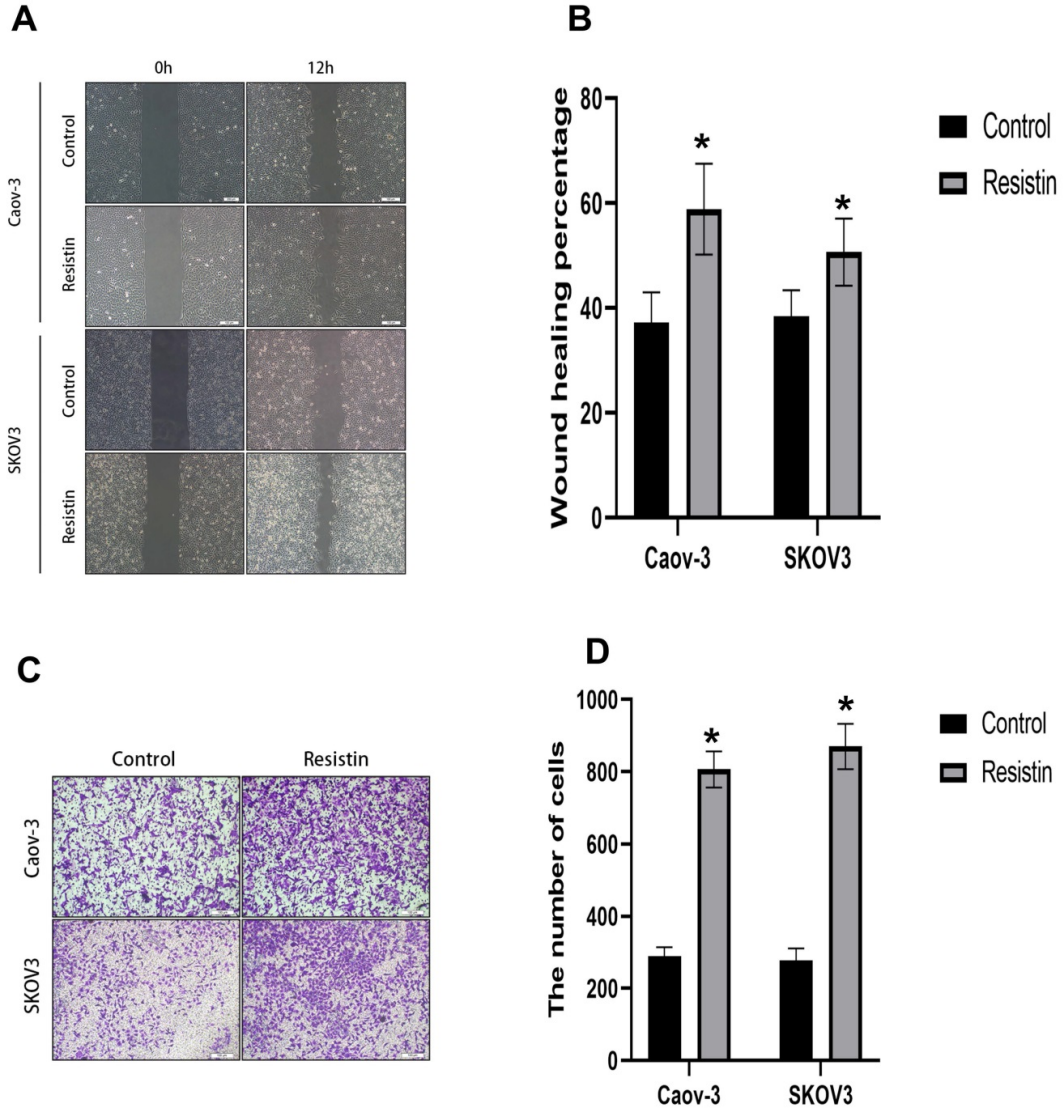
** The effect of resistin on the migration of SKOV3 and CAOV3 cells in ovarian epithelial carcinoma cells. (A, B)** Dose-response effect of 12 h resistin treatment on cell healing rate levels in SKOV3 and CAOV3 cells, Resistin promoted the SKOV3 and CAOV3 cell migration (*p<0.05). **(C, D)** Transwell migration assay. Quantification of migration cells with changes in resistin expression (*p<0.05).

Cell migration was evaluated by wound healing and Transwell assays. Cell mobility was also detected in the wound healing assay. SKOV3 or CAOV3 cells were grown to confluency, and resistin (40 ng) was added to induce cell healing. Scratch closure was monitored for 12 h. Microscopic images taken at 12 h post-scratching are shown in Figure 4. Resistin increased the migration rate of SKOV3 and CAOV3 cells (Figure 4A, 4B) (*P < 0.05). The Transwell migration assay showed that resistin promoted the invasive ability of SKOV3 and CAOV3 cells (Figure 4C, 4D) (*P < 0.05).

## Discussion

Several clinical and experimental studies have confirmed the correlation between obesity and cancer and shown that obese patients have a relatively higher risk of developing certain types of cancer 15. In addition, it has been reported that the metabolic effect of obesity, through insulin resistance, is a risk factor for cancer development. This evidence was obtained in epidemiological studies and reported that patients with metabolic syndrome have a higher incidence of cancer 16. Studies have also shown that young adults with higher BMI are at increased risk of premenopausal ovarian cancer, and the mortality rate of obese women with ovarian cancer is higher than that of women of normal weight. This is attributed to the secretion of hormones or proteins by the adipose tissue, which leads to the overgrowth of ovarian cancer cells 3.

In the tumor microenvironment, adipocytes are an important cell type. Histological studies showed that the interaction between tumor cells and adipocytes promotes changes in the tumor cell phenotype 17,18. Several clinical studies revealed that the amount of adipose tissue surrounding the tumor tissue is positively correlated with the prognosis of breast, prostate, pancreatic, renal, and colon cancer 19. Data from laboratory studies also indicate that adipocytes play an important role in maintaining the malignant phenotype of tumor cells. Adipocytes can promote the proliferation of breast and intestinal cancer cells 20,21. Ovarian cancer often spreads to distant organs either through direct spread or by pelvic and abdominal dissemination. At the time of ovarian cancer diagnosis, cancer cells have already spread to the omentum in 80% of patients. The most common site of metastasis is the human omentum. Therefore, we predicted that human omental adipocytes induce the proliferation and invasion of ovarian cancer cells *in vivo*. It has been suggested that the cells secreted by adipocytes attract cancer cells. Cancer cells on the greater omentum grew much faster than at the original lesion. Nieman et al. 22 found that omental fat cells can promote the location, migration, and invasion of ovarian cancer cells. It has also been speculated that adipocytes play an important role in the metastasis of ovarian cancer. Adipocytes in the microenvironment of ovarian cancer cells may produce some molecules that can help cancer cells maintain their malignant phenotype. Thus, investigating these molecules and their roles is crucial for identifying new treatment targets for ovarian cancer.

Resistin is a new adipocytokine involved in the insulin signaling pathway, the pathogenesis of osteoarthritis 23,24, and promoting the proliferation and migration of vascular smooth muscle cells 25. Multiple studies have shown that obesity-related serious diseases, such as cardiovascular disease and malignancies, are associated with elevated resistin levels 26. In addition, a meta-analysis suggested that resistin is an independent biomarker for the risk of obesity-related cancers. In patients with obesity-related cancers, circulating resistin levels were higher than those in normal controls 27.

Studies have shown that resistin expression is elevated in a variety of tumors. We analyzed the relationship between resistin expression and clinicopathological parameters in 50 EOC specimens by immunohistochemical analysis. We found that resistin expression was positively correlated with pathological grade and LN metastasis, regardless of age, histological type, residual tumor size, and clinical stage. The pathological grade of the tumor is an indicator of tumor malignancy. As resistin expression was found to be correlated with the pathological grade of the patient, it can be used to evaluate malignancy in EOC to a certain extent. Among the 50 EOC cases evaluated, 40 cases showed positive resistin expression (+ to +++), and 22 cases showed low differentiation. Among them, 8 cases were early ovarian cancer at clinical stages I or II. Thus, the expression of resistin was consistent with the high malignancy of the pathological grade.

Our data indicate that high expression of resistin in EOC contributes to the proliferation behavior of EOC and participates in its progression. However, the mechanism and influence of resistin on EOC progression requires further analysis.

We found that resistin expression was positively correlated with the pathological grade and LN metastasis. These data indicate that the expression of resistin contributes to more aggressive behavior of EOC. In addition, patients with high resistin expression showed shorter DFS and OS. Furthermore, we separately evaluated the prognostic values of various factors by univariate Cox proportional analysis and found that resistin expression was significantly associated with both DFS and OS. Based on the results of multivariant Cox proportional analysis, LN metastasis, resistin expression, and age were found to be independent factors for predicting EOC prognosis.

As a key intracellular molecule, mTOR can sense the energy levels inside and outside the cell to regulate protein transcription and translation, thereby regulating the survival, growth, proliferation and differentiation of a series of cells and tissues. This study showed that resistin promoted the proliferation of SKOV3 and CAOV3 cells in time- and dose-dependent manners. Furthermore, Immunoblotting was used to detect the changes in mTOR and its downstream target and phosphorylated protein (P70S6K) in SKOV3 and CAOV3 cells. We found that resistin may promote the proliferation of SKOV3 and CAOV3 cells by activating mTOR and P70S6K. In addition, rapamycin inhibited the activity of 70-KDaS6 kinase (P70S6K) downstream of mTOR, demonstrating that resistin promotes the proliferation of ovarian cancer cells by activating mTOR. Based on these findings, activating the mTOR signaling pathway may be a therapeutic target in patients who are obese and have ovarian cancer.

To further examine the effect of resistin on the progression of EOC, we treated two ovarian cancer cell lines, SKOV3 and CAOV3, with resistin. The results showed that the resistin-induced migration of SKOV3 and CAOV3 cells was significantly increased. In addition, the Transwell migration assay was used to determine the effect of resistin on the migration of SKOV3 and CAOV3 cells. The data showed that resistin significantly increased cell migration compared to the control group (*P* < 0.05). These findings suggest that resistin plays a key role in promoting the migration of SKOV3 and CAOV3 cells.

## Conclusion

Resistin was highly expressed in EOC tissue and was associated with poor prognosis in patients with EOC. These results suggest that the proliferation of EOC cells was mediated through the mTOR pathway, confirming the contribution of resistin to EOC migration. Overall, the results indicate that resistin plays a key role in the proliferation and migration of ovarian cancer cells and thus may be useful as a target for treating ovarian cancer. However, the exact mechanism and downstream signaling pathways remain unknown and require further analysis.

## Figures and Tables

**Table 1:** Relationship between resistin expression and clinicopathological characteristics in EOC

Clinicopathological characteristics	*n*	Resistin expression		χ^2^	*P*
		+	-		
**Age (y)**					
<55	19	16 (84.2)	3 (15.8)		
≥55	31	25 (80.6)	6 (19.4)	0.101	0.75
**Histological differentiation**					
G1	26	18 (69.3)	8 (30.8)		
G2/G3	24	22 (91.7)	2 (8.3)	5.682	0.017
**Histotype**					
Serous	18	14 (77.8)	4 (22.2)		
Mucinous	32	26 (81.3)	6 (18.7)	3.116	0.78
**FIGO staging**					
I, II	21	15 (71.4)	6 (28.6)		
III/IV	29	26 (89.7)	3 (10.3)	2.552	0.11
**LN metastasis**					
Negative	22	15 (68.2)	7 (31.8)		
Positive	28	26 (93.3)	2 (0.67)	4.023	0.045
**Residual tumor**					
<1 cm	32	26 (81.3)	6 (18.7)	0.034	0.854
≥1 cm	18	15 (83.3)	3 (16.6)		

*P < 0.05. **Abbreviations**: EOC, epithelial ovarian cancer; FIGO, International Federation of Gynecology and Obstetrics; LN, lymph node.

**Table 2:** Univariate and multivariate analyses of prognostic markers for OS in patients with EOC

	Univariate analysis		*P*	Multivariate analysis	95% CI	*P*
	HR	95% CI		HR		
Age (≥55 y)	2.132	1.243-3.825	0.021*	2.158	0.960-4.845	0.063
FIGO staging (II-IV)	1.539	0.9631-2.09	0.039*	1.456	0.403-5.269	0.567
Differentiation	5.473	1.530-17.981	0.006*	1.231	0.430-3.525	0.699
LN metastasis	0.913	0.912-0.962	0.001*	1.985	0.803-4.906	0.138
Residual tumor (≥1 cm)	2.115	1.153-3.753	0.034*	0.635	0.184-2.457	0.51
Resistin expression	2.753	1.467-5.321	0.002*	7.004	1.894-25.899	0.040*

*P < 0.05. **Abbreviations**: CI, confidence interval; EOC, epithelial ovarian cancer; FIGO, International Federation of Gynecology and Obstetrics; HR, hazard ratio; LN, lymph node; OS, overall survival.

**Table 3:** Univariate and multivariate analyses of prognostic markers for DFS in patients with EOC

	Univariate analysis			Multivariate analysis		
	HR	95% CI	*P*	HR	95% CI	*P*
Age (≥55 y)	1.653	0.934-2.702	0.049*	4.563	1.754-11.872	0.002*
FIGO staging (II-IV)	1.441	1.075-2.191	0.015*	2.271	0.619-8.375	0.216
Differentiation	4.693	1.827-11.899	0.001*	0.748	0.281-1.992	0.561
LN metastasis	1.325	0.853-2.718	0.022 *	3.23	1.183-8.823	0.022*
Residual tumor (≥1 cm)	2.865	1.583-4.784	0.035*	0.572	0.143-2.280	0.428
Resistin expression	1.893	1.106-3.364	0.023*	10.851	2.691-43.745	0.001*

*P < 0.05. **Abbreviations**: CI, confidence interval; DFS, disease-free survival; EOC, epithelial ovarian cancer; FIGO, International Federation of Gynecology and Obstetrics; HR, hazard ratio; LN, lymph node.
